# Functional near-infrared-spectroscopy-based measurement of changes in cortical activity in macaques during post-infarct recovery of manual dexterity

**DOI:** 10.1038/s41598-020-63617-0

**Published:** 2020-04-15

**Authors:** Junpei Kato, Toru Yamada, Hiroshi Kawaguchi, Keiji Matsuda, Noriyuki Higo

**Affiliations:** 1 Human Informatics and Interaction Research Institute, National Institute of Advanced Industrial Science and Technology, Tsukuba Ibaraki, 305-8568 Japan; 2 Graduate School of Comprehensive Human Sciences, University of Tsukuba, Tsukuba Ibaraki, 305-8577 Japan

**Keywords:** Biophotonics, Imaging and sensing, Stroke, Rehabilitation, Motor control, Stroke

## Abstract

Because compensatory changes in brain activity underlie functional recovery after brain damage, monitoring of these changes will help to improve rehabilitation effectiveness. Functional near-infrared spectroscopy (fNIRS) has the potential to measure brain activity in freely moving subjects. We recently established a macaque model of internal capsule infarcts and an fNIRS system for use in the monkey brain. Here, we used these systems to study motor recovery in two macaques, for which focal infarcts of different sizes were induced in the posterior limb of the internal capsule. Immediately after the injection, flaccid paralysis was observed in the hand contralateral to the injected hemisphere. Thereafter, dexterous hand movements gradually recovered over months. After movement recovery, task-evoked hemodynamic responses increased in the ventral premotor cortex (PMv). The response in the PMv of the infarcted (i.e., ipsilesional) hemisphere increased in the monkey that had received less damage. In contrast, the PMv of the non-infarcted (contralesional) hemisphere was recruited in the monkey with more damage. A pharmacological inactivation experiment with muscimol suggested the involvement of these areas in dexterous hand movements during recovery. These results indicate that fNIRS can be used to evaluate brain activity changes crucial for functional recovery after brain damage.

## Introduction

Neuronal motor systems have the capacity for functional recovery following brain damage such as that induced by stroke, and functional recovery can be enhanced by postlesion rehabilitative training^1,2^. Compensatory activity changes in the brain areas that remain undamaged are thought to underlie functional recovery^1,3–16^. Therefore, it is important to monitor brain activity during rehabilitative training to determine whether the training is actually inducing appropriate brain activity changes. Clinical studies in human stroke patients, as well as experimental studies in animals in which damage is artificially induced in a specific region in the brain, have helped to elucidate the compensatory changes of brain activity that occur during the course of functional recovery^1,7–10,17^.

We previously established a macaque model for inducing artificial damage in brain regions involved in the transduction of motor commands^2,18,19^. The motor cortex and corticospinal tract of macaque monkeys are more comparable to those of humans than are those of other animals used in experimental research (see ref. ^20^. for review). This motor system homology with that of humans, in combination with the relatively large macaque brain, makes imaging data obtained in macaque monkeys comparable to data obtained in clinical research. Therefore, studying macaque models of brain damage can facilitate the translation of findings to stroke patients.

Brain damage related to hand functionality can greatly affect the quality of human life and is therefore an important area of study. Using H_2_^15^O-positron emission tomography (PET) scans in macaque monkeys, we previously showed that the ipsilesional ventral premotor cortex (PMv) is involved in the recovery of dexterous hand movements after lesioning of the primary motor cortex (M1)^21^. Enhanced involvement of the PMv was also observed with functional magnetic resonance imaging (fMRI) of stroke patients during recovery of hand movements^12,14^, indicating that this area is a candidate for assessing the effectiveness of rehabilitative training for hand motor dysfunction after brain damage. PET scanning is, however, not appropriate for monitoring brain activity in clinical rehabilitation because of the invasiveness of using radioactive tracers. Moreover, the subject needs to be fixed in the scanner during PET scanning and fMRI, and their natural posture and movements, such as those used during rehabilitative training, are therefore restricted. Functional near-infrared spectroscopy (fNIRS) offers potential for recording in unrestrained subjects, including those performing rehabilitative training, although it is essential to remove artifacts originating from body motions and optode fluctuations. Previous studies using fNIRS in stroke patients have reported compensatory changes of brain activity compared with that in normal subjects (see ref. ^22–24^. for review). Experimental studies using lesioned animal models will allow the brain activity changes caused by rehabilitative training to be evaluated more clearly, because brain activity after functional recovery can be compared with that measured before brain damage in the same subjects. Moreover, the causal role of the brain areas where activity changes are observed in functional recovery can be confirmed in animal models by the use of invasive pharmacological inactivation.

In a previous study^25^, we developed an fNIRS system for monitoring macaque cerebral motor activity during voluntary movements without head fixation. We recently used this system to successfully monitor functional hemodynamic responses in motor-related cortical areas reproducibly over long periods of time. Here, we used this system to investigate whether brain activity changes during functional recovery after brain damage could be evaluated by fNIRS.

**Figure 1:**
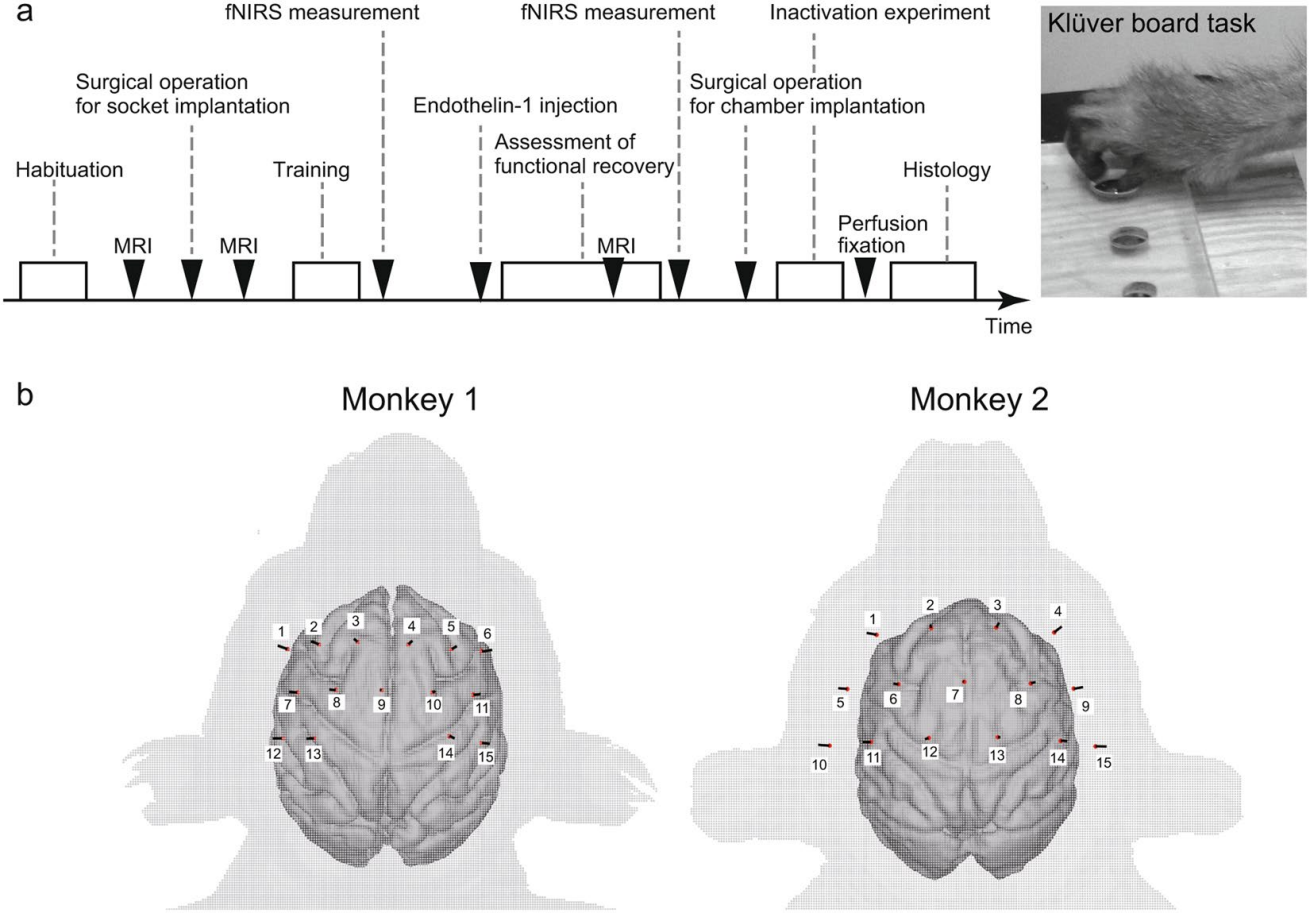
Experimental design and placement of optodes. (**a**) The experimental design. After implantation of optode sockets, the animals were trained on the Klüver board task (shown in the image at the right) and the vertical slit task. We first measured hemodynamic responses evoked during the Klüver task with fNIRS before infarction. Focal infarcts were then induced by local injection of endothelin-1 into the posterior limb of the internal capsule. After the recovery of dexterous hand movements with postlesion motor training, the hemodynamic response evoked during a small-object retrieval task (Klüver board or vertical slit) was again measured with fNIRS. A pharmacological inactivation experiment was performed by using muscimol, a gamma-aminobutyric acid receptor agonist. MRI scans were performed to decide on and confirm the arrangement of optode sockets, to decide on the stereotaxic coordinates for the endothelin-1 injection, and to evaluate the size of the infarcts. Finally, the brain damage caused by the infarcts and the locations of the muscimol injections were confirmed histologically in Nissl-stained brain sections. (**b**) The arrangement of fNIRS optodes on the skull surfaces of the monkeys.

We first measured the hemodynamic responses evoked during a small-object retrieval task before infarction (Fig. 1a). Focal infarcts were then induced in the posterior limb of the internal capsule by using a technique we developed^18^. The internal capsule carries the corticospinal tracts, and the severity and outcome of motor impairments depend on the degree of damage to this area. After the recovery of dexterous hand movements with postlesion motor training, the hemodynamic response evoked during a small-object retrieval task was measured again. In addition to the fNIRS measurements, another experiment was performed in which we pharmacologically inactivated the region where hemodynamic responses increased during functional recovery by using muscimol, a gamma-aminobutyric acid receptor agonist. We confirmed that local inactivation of the active regions identified with fNIRS measurement reversed the recovery of hand movements in the small-object retrieval task.

## Results

### Hemodynamic response before infarction

**Figure 2:**
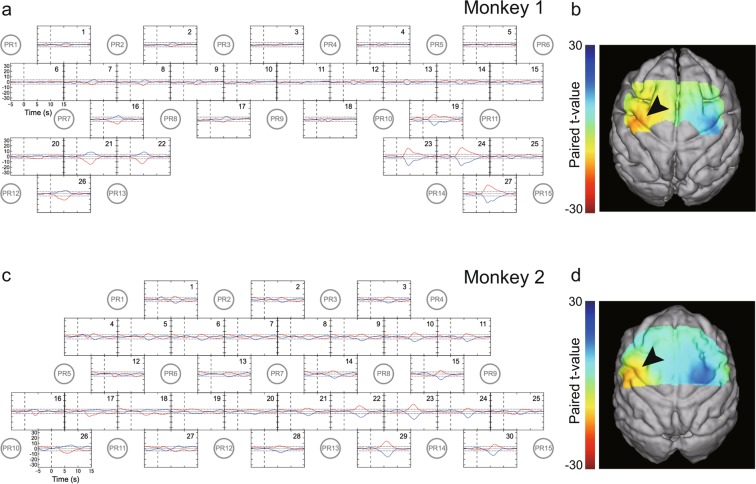
Hemodynamic responses before infarction. (**a,c**) Time-course curves of paired *t*-values of hemodynamic response in Monkeys 1 (**a**) and 2 (**c**) before infarction. The *t*-values for the paired *t*-test were calculated from the difference in hemodynamics during trials of left-hand and right-hand use in the session, using a food well with a diameter of 10 mm. The *t*-values of changes in oxygenated (ΔHbO) and deoxygenated (ΔHbR) hemoglobin in each channel (indicated by the numbers in each frame) are drawn with red and blue lines, respectively. The horizontal dotted lines in each frame indicate levels of *p* = 0.0001. The horizontal axis indicates time from the onset of the retrieval movement (vertical dashed line). The locations of the fNIRS optodes are indicated by gray circles with probe numbers (PR). (**b,d**) Color-mapping of *t*-values onto the anatomical MRIs for Monkeys 1 (**b**) and 2 (**d**). The *t*-values for ΔHbO around 5.0 s (for Monkey 1) and 5.5 s (for Monkey 2) after task onset are depicted. Amplitudes of *t*-values at most channels were maximal at this time. Arrowheads indicate the positions of the “hand knob” in M1 in the left hemisphere.

Before infarction, both ΔHbO and ΔHbR were evoked during the small-object retrieval task (Klüver board). The hemodynamic responses were similar between the two monkeys and consistent with our previous report^25^; ΔHbO and ΔHbR exhibited opposite directional changes, and larger increases in ΔHbO and decreases in ΔHbR were recorded at channels in the hemispheres contralateral to the hand being used. As in the previous study, paired *t*-tests were conducted for each Hb species for each channel in each session to statistically evaluate the difference in Hb changes between use of the left and right hands (Fig. 2a,c). Color-mapping of *t*-values (at the time when the amplitudes of the *t*-values at most channels were maximal) onto the anatomical MR image for each monkey indicated that channels with highly significant *t*-values in ΔHbO were distinctively localized around the hand area of M1 (Fig. 2b,d).

### Functional recovery after infarction

Before endothelin-1 injection, monkeys performed the Klüver board and vertical slit tasks smoothly with dexterous hand movements that included a precision grip, holding the morsel between the tips of the index finger and thumb (Supplementary Video S1). Immediately after endothelin-1 injection into the posterior internal capsule of the left hemisphere, motor paralysis was observed in the contralateral (right) forelimb. The macaques showed impaired performance in the small-object retrieval tasks and no precision grip in the right hand during the first month after the injection.

**Figure 3:**
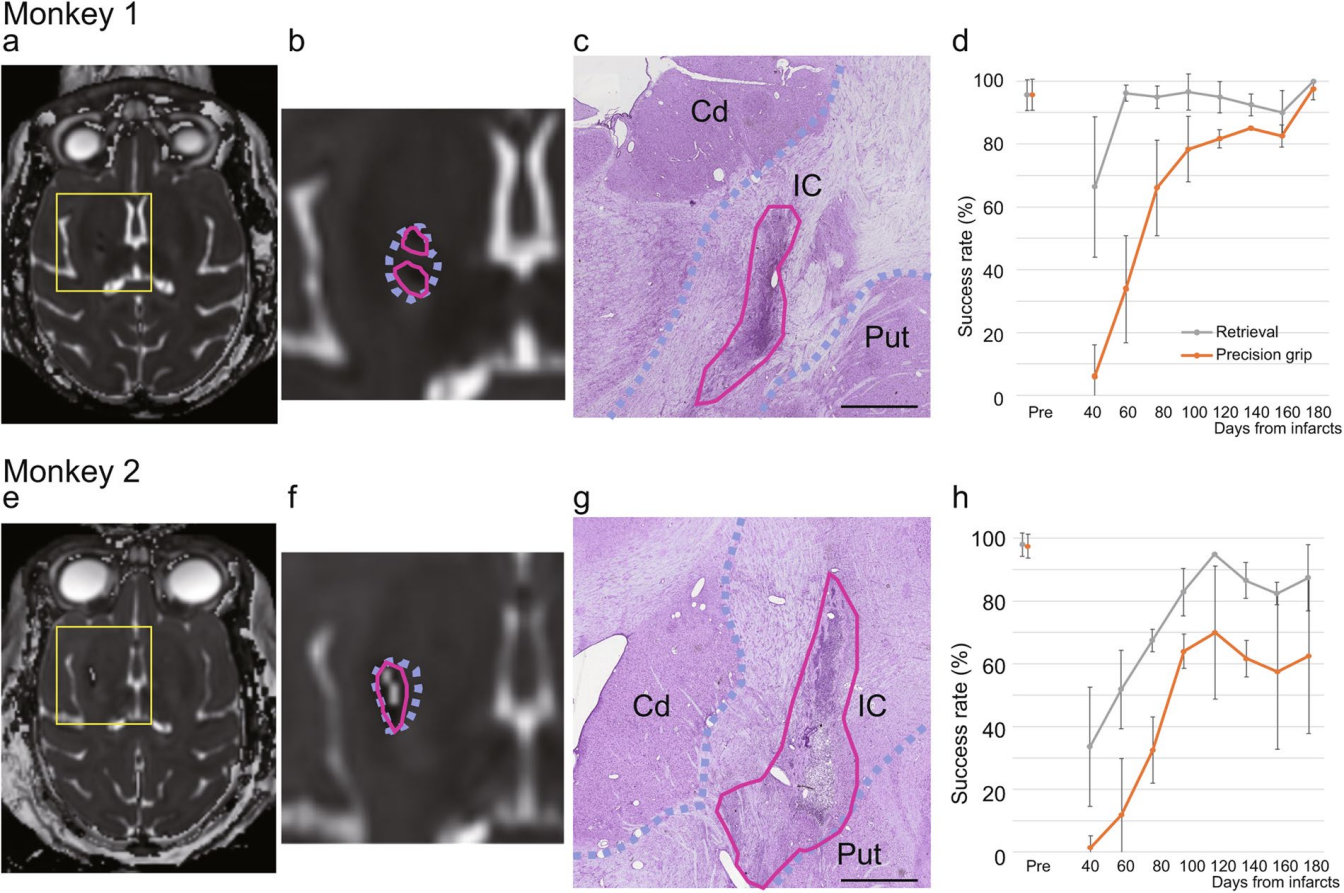
Extent of infarcts and behavioral recovery. (**a,e**) Axial T2-weighted MR images of the brains of Monkeys 1 (**a**) and 2 (**e**), showing the locations of the infarcts 1 month after endothelin-1 injection. The yellow squares indicate the areas shown in (**b,f**). (**b,f**) Higher-magnification MR images around the infarcts. The violet dotted lines indicate the anterior two-thirds of the posterior limb of the internal capsule, which contains fibers of the corticospinal tract. The magenta solid lines indicate the hypointense infarct core; T2-hyperintense cystic lesions were also found in Monkey 2. (**c,g**) Nissl-stained coronal sections made from the anterior two-thirds of the posterior limb of the internal capsule after the completion of the fNIRS measurements and pharmacological inactivation experiment. The area bordered by the violet dotted lines indicates the internal capsule (IC). The magenta solid lines indicate the lesioned area, which is defined as the area of a dense concentration of small cells (5–10 μm in diameter), which presumably include both glial cells and blood cells. The images were acquired with a microscope (BX60, Olympus, Tokyo, Japan) equipped with a 3CCD color video camera (DHC-950, Sony, Tokyo, Japan) and digitized with an image analysis system (version 2019.1.1, Stereo Investigator, MBF Bioscience Inc., Williston, VT, USA. URL https://www.mbfbioscience.com/stereo-investigator.). Cd, caudate nucleus; Put, putamen. Scale bar = 1 mm. (**d,h**) Time course of changes in the success rates of retrieval (gray) and precision grip (orange), as evaluated with the vertical slit task. The mean and standard deviation before infarction and every 20 days after infarction are indicated.

The areas of infarction 1 month after injection (Fig. 3a,e, magenta solid line in Fig. 3b,f) included the anterior two-thirds of the posterior limb of the internal capsule (violet dotted line in Fig. 3b,f), where the motor tracts from the M1 hand area descend. The infarcted area occupied most of the anterior two-thirds of the posterior limb of the internal capsule in Monkey 2 (Fig. 3f), whereas there was an area that was spared from infarction in Monkey 1 (Fig. 3b). Thus, the infarction volume within the internal capsule in Monkey 2 was larger than that in Monkey 1 (Table 1). This was probably resulted from a difference in the backflow of endothelin-1 through the needle tracks, as described in our previous study^18^. The enduring brain damage was also confirmed histologically in Nissl-stained brain sections after perfusion fixation (Fig. 3c,g), and the histological lesion in Monkey 2 was larger than that in Monkey 1.

**Table 1:** Characteristics of monkeys used in the study, and timing of experiments.

	Monkey 1	Monkey 2
Weight (kg)	5.0	6.8
Age (years)	8.2	9.9
Sex	Female	Female
Infarct volume (mm^3^)	91.2	117.6
Infarct volume within internal capsule (mm^3^)	38.4	117.6
Duration of recovery (time taken for retrieval success rate to plateau, days)	60	100
Recovery of precision grip at plateau (% preinfarction value)	90.7	64.4
Time of fNIRS measurement after infarction (days)	92	142
Time of inactivation experiment after infarction (days)	152–171	163–203

The success rate of retrieval, as evaluated with the vertical slit task, increased during the 2 to 3 months after the injection in both monkeys (Fig. 3d,h, see Supplementary Video S1). The use of a precision grip in the vertical slit task of both monkeys also increased after the first month. In Monkey 1, the success rate at 6 months after the injection was within the 95% confidence level for performance before the injection (Fig. 3d). Monkey 2 also showed significant recovery in hand movements after the first months following infarction, but its success rate plateaued at about 4 months, and at 6 months after the injection it was below the 95% confidence level of the pre-injection rate (Fig. 3h).

### Hemodynamic response after recovery of hand movements

**Figure 4:**
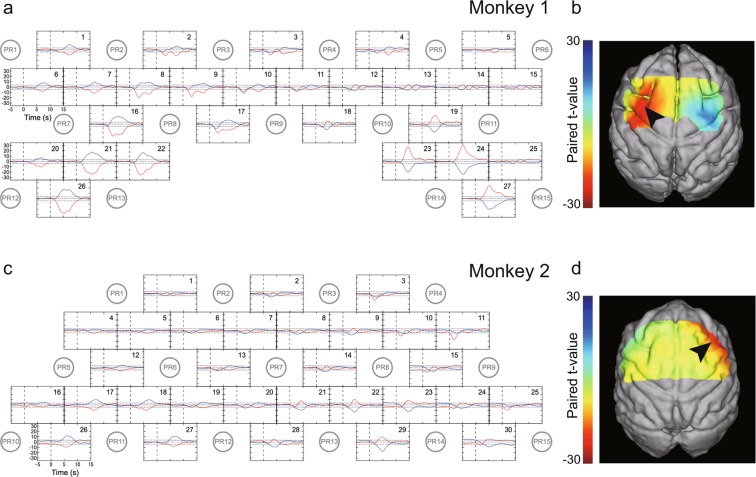
Hemodynamic changes with hand use after recovery. (**a,c**) Time-course curves of paired *t*-values of the hemodynamic response in Monkeys 1 (**a**) and 2 (**c**) after recovery of dexterous hand movements after infarction. The *t*-values for the paired *t*-test were calculated from the difference in hemodynamics during trials of left-hand and right-hand use in the session using a food well with a diameter of 10 mm. The *t*-values of ΔHbO and ΔHbR in each channel are drawn with red and blue lines, respectively. The horizontal dotted lines in each frame indicate levels of *p* = 0.0001. The horizontal axis indicates time from the onset of the retrieval movement (vertical dashed line). The locations of fNIRS optodes are shown by gray circles with probe numbers (PR). (**b,d**) Color-mapping of *t*-values onto the anatomical MR images of Monkeys 1 (**b**) and 2 (**d**). The *t*-values for ΔHbO around 3.5 s (for Monkey 1) and 2.0 s (for Monkey 2) after task onset are depicted. Amplitudes of *t*-values at most channels were maximal at these times. Arrows indicate the positions of the PMv in the left (**b**) and right hemispheres (**d**).

fNIRS measurement was again conducted for each monkey after sufficient recovery in retrieval performance (>90% of prelesion success rates of retrieval, i.e., 3 and 4 months after injection for Monkeys 1 and 2, respectively). Although hemodynamic responses were evoked by hand movements during the small-object-retrieval task after recovery, their spatiotemporal patterns were different from those before infarction and, moreover, differed between the two monkeys. In Monkey 1, the hemodynamic response was enhanced not only in the M1 hand area but also in cortical areas located rostrally to M1 in the ipsilesional (left) hemisphere, including the PMv (Fig. 4a,b). By contrast, in Monkey 2, the hemodynamic response was prominently enhanced in the PMv of the contralesional (right) hemisphere rather than the ipsilesional (left) hemisphere (Fig. 4c,d).

**Figure 5:**
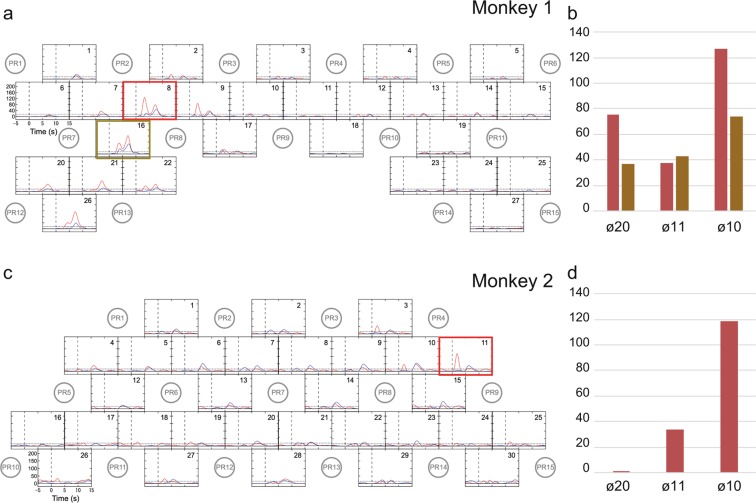
Interaction effects between infarction and hand. (**a,c**) Two-way MANOVA was applied to the data presented in Figs. 2 and 4 to find interaction effects between infarction (Pre/Post) and the hand used (Left/Right). Time-course curves of the interaction effects in Monkeys 1 (**a**) and 2 (**c**) are shown. The F-values of ΔHbO and ΔHbR in each channel are drawn with red and blue lines, respectively. The dotted lines in each frame indicate levels of *p* = 0.0001. The horizontal axis indicates time from the onset of the retrieval movement (vertical dashed lines). Locations of fNIRS optodes are shown by gray circles with probe numbers (PR). The red-highlighted frames indicate sites with high interaction effects, which we selected for muscimol injection. (**b,d**) Changes of the interaction effect depending on the size of the food well at channels 8 (red) and 16 (gold) in Monkey 1(**b**) and at channel 11 in Monkey 2 (**d**).

We applied two-way MANOVA to find interaction effects between infarction (pre/post) and the hand used (left/right). In Monkey 1, statistically significant F-values were found in ipsilesional (left) hemisphere channels 20, 21, 22, and 26, which corresponded to M1; channels 1,7, 8, and 16, which corresponded to the PMv; and channels 9 and 17, which corresponded to the dorsal premotor cortex (PMd) (Fig. 5a). Among these, the enhancements in channels 8 and 16 within the PMv were most pronounced. We evaluated the effect of food-well size on the changes of F-values in channels 8 and 16. In both channels, statistically significant F-values were found for wells of all three sizes, with the highest value observed for the ø10-mm well (Fig. 5b). In Monkey 2, statistically significant but low F-values were found in ipsilesional (left) hemisphere channels 18, 19, and 27, which corresponded to M1, and channels 1,4, 5, and 6, which corresponded to the PMv (Fig. 5c). Much higher F-values were found in channel 11, which corresponded to the PMv of the contralesional (right) hemisphere, and the neighboring channels. The F-value in channel 11 was higher for the ø10-mm well than for the ø20- and ø11-mm wells (Fig. 5d).

In both monkeys, the peak time of the hemodynamic responses in the PMv areas—channels 8 and 16 of Monkey 1 and channel 11 of Monkey 2 (Table 2)—also changed. Before infarction, the peak time of the paired *t*-values in the PMv areas was slightly earlier than that in the hand area of M1 (channels 21 and 22 of Monkey 1 and channels 17 and 18 of Monkey 2), but the difference was not statistically significant (*p* > 0.05, Tukey–Kramer test after one-way ANOVA, Table 2). The peak time of hemodynamic response in the PMv areas after recovery from infarction was significantly earlier than that in either M1 or the PMv before infarction (*p* < 0.01); it was also significantly earlier than that in M1 after recovery (*p* < 0.05 and *p* < 0.01 in Monkeys 1 and 2, respectively).

**Table 2:** Peak times of paired *t*-values of hemodynamic responses after task onset (mean and standard deviation, in seconds) in Monkeys 1 and 2.

		Before infarction	After recovery
Monkey 1	PMv	5.02 ± 0.31	3.42 ± 0.54
	M1	5.09 ± 0.38	4.26 ± 0.65
Monkey 2	PMv	4.03 ± 1.26	1.78 ± 0.16
	M1	5.31 ± 0.49	4.97 ± 0.83

### Effects of inactivation

**Figure 6:**
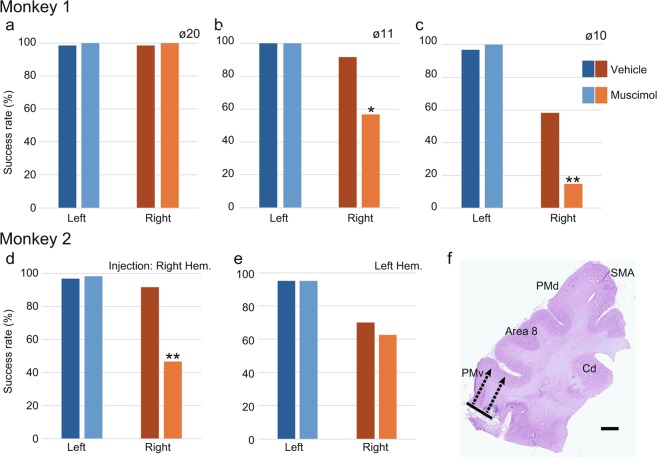
Effects of inactivating PMv on the performance of dexterous hand movements. (**a–c**) Muscimol injections into the ipsilesional (left) PMv of Monkey 1 resulted in a significant deficit of the right hand in performing the Klüver board task with well diameters of 11 or 10 mm, but not 20 mm. (**d,e**) Muscimol injections into the contralesional (right) PMv of Monkey 2 resulted in a significant deficit of the right hand in performing the vertical slit task (**d**), whereas muscimol injections into the ipsilesional (left) PMv did not significantly change performance for either hand (**e**). *p < 0.05, **p < 0.01, Fisher’s exact test. (**f**) Representative Nissl-stained section showing brain microinjection sites in the contralesional PMv of Monkey 2. The dotted lines with arrows indicate the estimated spread of muscimol. The muscimol injection was also confirmed in the ipsilesional PMv of both monkeys in the same manner. The image was acquired with a microscope (BX60, Olympus, Tokyo, Japan) equipped with a 3CCD color video camera (DHC-950, Sony, Tokyo, Japan) and digitized with an image analysis system (version 2019.1.1, Stereo Investigator, MBF Bioscience Inc., Williston, VT, USA. URL https://www.mbfbioscience.com/stereo-investigator.). Scale bar = 2 mm.

Enhanced task-evoked hemodynamic responses were found in the PMv areas. To investigate the potential involvement of these areas in recovery of hand movements, they were transiently inactivated with a focal microinjection of muscimol. In Monkey 1, we injected muscimol into the middle point between channels 8 and 16, which corresponded to the ipsilesional (left) PMv where the most prominent enhancement in activation was observed. The muscimol injections did not have a significant effect on performance on the Klüver board task when the well diameter was 20 mm (Fig. 6a), but it resulted in a significant deficit of the right hand when the well diameter was 11 or 10 mm, compared with the performance after injection of an equivalent volume of vehicle (Fig. 6b,c). In Monkey 2, muscimol injections into channel 11, which corresponded to the contralesional (right) PMv, resulted in a significant deficit of the right hand in performing the vertical slit task as compared with the performance after vehicle injection (Fig. 6d). By contrast, muscimol injections into the ipsilesional (left) PMv, just opposite channel 11 across the midline, resulted in a slight but not significant deficit of the right hand in performing the vertical slit task (Fig. 6e). No deficit was observed in the left hand in any experimental condition in either monkey. Locations of the muscimol injections in the PMv were confirmed histologically in Nissl-stained brain sections after completion of the pharmacological inactivation experiments (Fig. 6f).

## Discussion

Using fNIRS allowed us to demonstrate robust changes in the task-evoked hemodynamic response after infarction. After the recovery of hand movements, task-evoked hemodynamic responses increased in areas within the PMv. This result is consistent with our previous study using PET scans in macaque monkeys, which showed that the PMv is involved in the recovery of hand movements after lesioning of M1^21^. The involvement of PMv during functional recovery of hand movements has been reported in other studies of brain-damaged monkeys^9,10,26,27^ and in stroke patients^12,14^. Our current results, in which activation was enhanced more in the ipsilesional PMv of the monkey with less damage (Monkey 1) and in the contralesional PMv of the monkey with more damage (Monkey 2), are also consistent with data from stroke patients and brain-damaged animals. Previous studies have indicated that functional reorganization of the motor cortex may occur in both the ipsilesional and contralesional hemispheres during functional recovery after stroke, and that the contralesional motor cortex may play a greater role in recovery when damage is more severe, because neuronal reorganization for functional recovery within the ipsilesional side may be difficult to accomplish under these circumstances^3–6,11,13,15,16,28^. In our previous PET study using M1-lesioned monkeys, enhanced activation was found in the ipsilesional PMv^21^; by 2 months after lesioning, the success rate of precision grip in the M1-lesioned monkeys had recovered to within the 95% confidence level for performance before the injection. This shorter period required for performance recovery than the 6 months needed for Monkey 1 indicates that the M1-lesioned monkeys in our previous study had less-severe brain damage than Monkey 1 in our present study. Involvement of the ipsilesional PMv has been reported by other studies, in which a partial lesion was made in the M1 of monkeys^9,10,26,27^. Therefore, the present results for Monkey 1 are consistent with those of the previous studies:^9,10,21,26,27^ activation of the ipsilesional PMv was enhanced during recovery of hand movement after mild brain damage. Further analysis with additional monkeys is needed to elucidate the factors that determine the hemisphere involved in functional recovery after unilateral brain damage.

Here, we found statistically significant enhancements of the hemodynamic response in M1 and the PMd, in addition to the PMv, after recovery from infarction. These results confirmed previous findings that all three of these regions are involved in motor recovery after stroke^6,17,29,30^. However, the hemodynamic responses showed much greater enhancement in the PMv than in M1 and the PMd, possibly because the PMv is involved in grasping and manipulating objects^31,32^, so that the small-object retrieval task specifically activates this region. It will be important to address the contributions of M1 and the PMd to further elucidate the mechanisms underlying motor recovery after infarction in the posterior internal capsule. Moreover, the supplementary motor cortex (SMA) plays a role in motor recovery after brain damage^33–36^. However, our two-way MANOVA analysis did not indicate very notable differences between the pre-lesion and post-recovery conditions in the channels above the SMA (channels 3, 10, and 11 in Monkey 1 and channels 2, 7, and 8 in Monkey 2; Fig. 5). Because most of the SMA is located on the midline sagittal surface of the hemispheres and its distant position from the head surface limits access of the light, fNIRS signal sensitivity at the SMA may be lower than that at the PMv. This may be why we failed to detect a functional difference at the SMA after motor functional recovery. In such cases, the use of a 3D reconstruction technique^37^, whereby there is compensation for sensitivity differences caused by differences in signal depth, may help to detect activation at deeper cerebral regions. Further analyses using such techniques may reveal the roles of both the SMA and the PMv in motor functional recovery. It is also important to identify the subcortical structures that, after infarction of the posterior internal capsule, are involved in motor recovery in concert with the intact motor cortical areas, as suggested by previous studies of motor recovery after unilateral M1 lesioning^38–40^.

Although the results of our fNIRS analysis using orthodox statistical procedures such as the paired *t*-test and MANOVA suggested that there was spatial reorganization of functional roles among the motor-related cortical regions, such analyses have limitations for revealing the causal relationships among these regions. Recent human studies using fMRI have used dynamic causal modeling to analyze changes in causal relationships among the motor-related cortical regions during motor functional recovery as changes in effective connectivity^41^. Similarly, in our monkey study, effective connectivity analysis may have been useful for investigating causal relationships; furthermore, the higher temporal resolution of fNIRS than of fMRI promises more granular analyses of effective connectivity analysis and Granger causality analysis. A further study combining these analytical techniques and fNIRS may shed more light on the mechanism of functional recovery.

In both monkeys after recovery, hemodynamic responses in the PMv had changed in both strength and timing. The peak *t*-values in both M1 and the PMv occurred significantly earlier after recovery from infarction than before infarction, and the post-recovery peak was earlier in the PMv than in M1 (see Table 2). It is not clear why these changes in response timing occurred in PMv. In a monkey model of M1 lesioning and motor recovery, our laboratory recently found that connections from the PMv to subcortical structures were rewired^40^, suggesting that indirect projections from the PMv to the spinal cord by way of subcortical structures may be recruited to send motor commands from the cerebral cortex. Motor command transmission via these indirect projections probably takes more time than transmission via the direct corticospinal projection for two reasons: relay along multisynaptic rather than monosynaptic pathways, and less-heavy myelination of the pathways^42,43^. It is likely that indirect projections from the PMv are also recruited during recovery from internal capsule infarction; therefore, early activation of the PMv may occur to compensate for the slow transmission of motor commands and to cooperate with motor command transmission via spared direct corticospinal projections. The more distinctive shortening of the time to peak *t*-value in the contralesional PMv of Monkey 2 than in the ipsilesional PMv in Monkey 1 supports this hypothesis, because the pathway recruited would be longer in the former than the latter, although the difference in peak time may be far larger than the difference in time required for neural transmission.

A clear limitation of our study was the small number of monkeys (n = 2); further analysis with a larger sample size is needed to determine how the changes in response timing are related to the size of the infarcts. Despite the small sample size, we were able to detect such changes in response timing because fNIRS has greater time resolution than fMRI and PET. Another limitation was that the functional assessment with fNIRS and the reversible inactivation were performed at a single time point during the plateau phase of motor recovery. It is possible that different areas may play a role at other time points—in particular during the earlier phase preceding the plateau, as suggested in previous brain imaging studies^21,44,45^. Even with these limitations of our study, we believe that fNIRS measurement is useful for evaluating the brain activity changes that are crucial for functional recovery after brain damage.

## Methods

### Subjects and study design

Two Japanese macaque monkeys were used (Table 1). The monkeys were older than 5 years and were purchased from a local provider. Monkey 1 was the same as the one used in our previous study^25^, in which the hemodynamic response in the intact motor-related cortical areas was measured with the fNIRS system described below. The animal-use protocol was approved by the Institutional Animal Care and Use Committee of the National Institute of Advanced Industrial Science and Technology (AIST) and was implemented in accordance with the “Guide for the Care and Use of Laboratory Animals” (eighth ed., National Research Council of the National Academies). The monkeys were housed as described in our previous study^21^; they were housed in adjoining individual primate cages (width, 750 mm; length, 950 mm; height, 930 mm) that allowed social interaction under controlled conditions of humidity, temperature, and light; they were monitored daily by the researchers and animal care staff to ensure their health and welfare. The housing area was maintained on a 12-h light/12-h dark cycle, and all experiments were conducted during the light cycle.

### fNIRS measurement

In our previous study^25^, a high-spatial-density fNIRS measurement system was developed for monitoring cerebral functional hemodynamic responses in monkeys. Its measurement reproducibility was confirmed in observations of functional hemodynamic responses in motor-related cortical areas of Monkey 1 in the intact condition. There, fNIRS optodes were directly affixed to the skull surface of the macaque both to ensure accurate absolute spatial placement of the optodes and to eliminate physiological signals originating from sources other than the brain. The possibility of signal contamination from tissues other than the scalp was also considered. Diploic veins are present in the skull itself; however, it is still unknown whether the blood flow in these veins changes upon task execution. Our previous study using the same experimental setup as in the present study revealed a significantly high correlation between ∆HbO and ∆HbR in almost all channels^25^. This strongly suggests that the hemodynamic response measured by our present system is caused by neurovascular coupling, because other hemodynamics—including those in the diploic veins—would reflect neither regional oxygen consumption nor regional washout of HbR by overcompensation of HbO. We applied the hemodynamic modality separation method^46^, which separates the fNIRS signal into a cerebral functional component and a systemic physiological component, to our experimental data. The separated systemic physiological component in each channel was very small and was not correlated with its original fNIRS signal (data not shown). On the basis of these findings, we conclude that signal contamination from blood in the diploic veins was negligible in the fNIRS data that we obtained.

The procedure used in the present study was almost identical to that of the previous study^25^. Briefly, the positions of the central and arcuate sulci were determined by using stereotaxic coordinates from T1- and T2-weighted MR images of each monkey’s head, obtained with a 3.0-T MRI system (Philips Ingenia 3.0 T, Philips Healthcare, Best, The Netherlands) (Fig. 1a). This anatomical information was used to determine the arrangement of optodes that would cover most of the motor-related cortical areas (Fig. 1b). Under pentobarbital anesthesia, the scalp was incised, and optode sockets were formed on the skull surface with self-curing acrylic resin (UNIFAST II Clear, GC Corporation, Tokyo, Japan) mixed with titanium oxide (KA-30, Titan Kogyo, Ltd., Ube, Japan) at a weight ratio of 1:450 to match its optical scattering property to that of the skull. A custom-made connector system was used for Monkey 1^25^, whereas the FC-type connector system was used for optode fixation in Monkey 2 for easier attachment. FC-type socket wells made of polyacetal were arranged on the skull surface and covered with resin. T1-weighted MR images of each monkey head with the optode sockets filled with Cu-sulfate solution (10 mM/L) were obtained to identify the position of each optode. The optical partial path length in the cortical layer at every measurement channel was calculated with a computer simulation of light propagation in monkey head models containing the sophisticated anatomical structure from both the T1 and T2 MR images. The optodes were fixed into the socket wells just before every experimental session. To detect fNIRS signals with a high spatial density of several millimeters in the inter-channel interval, a triangular bidirectional measurement was implemented, as described in our previous study^25^.

The fNIRS measurements were obtained as the monkeys performed a small-object retrieval task, the Klüver board task (Fig. 1a), closely resembling that used in our previous studies^2,47^. In this task, the monkey sat in a primate chair and retrieved a small, spherical food pellet (5 mm in diameter) from the Klüver board, which contained cylindrical wells of three different sizes (10, 11, and 20 mm in diameter, 5 mm in depth). The size of the well was fixed within a daily session, and the monkey retrieved food pellets approximately every 20 s by using each hand alternately. One hundred fifty trials (75 for each hand) were performed in each daily session. The onset of a food-retrieval movement by either the left or right hand was detected by a digital laser sensor (LV-11SB with sensor head LV-S72, Keyence, Osaka, Japan), which was fixed at the slit in front of the food well. The onset was defined as the time point when the sensor detected the monkey’s upper limb. From the recorded video data, we confirmed that the period of time between this onset and the start of digital flexion differed among most trials within only several tens of milliseconds.

### Infarct induction and behavioral tests

Focal infarcts were induced in the posterior limb of the internal capsule by using the same procedure as in our previous work^18^. A craniotomy was made over the internal capsule, and endothelin-1 (1.5 μg/μL, 4198-v, Peptide Institute, Inc., Osaka, Japan)—a vasoconstrictor peptide^48^—was then injected into the internal capsule via a microsyringe (MS-25, Ito Corporation, Fuji, Japan). To evaluate the size of the infarcts in each monkey, MRI scans were performed 1 month after the injection; the area of infarcts was seen as a hypointense core with a surrounding hyperintense rim on T2-weighted MR images. The unbiased volumes of the infarcts were calculated on the basis of Cavalieri’s principle^49^ by using StereoInvestigator imaging software (version 2019.1.1, MBF Bioscience, Williston, VT, USA. URL https://www.mbfbioscience.com/stereo-investigator.).

Performance of hand movements before and after the endothelin-1 injections was evaluated by means of the vertical slit task, a small-object-retrieval task performed in the primate cage and identical to that used in our previous studies^2,18,47^. In the test session with this task, the macaques retrieved a small piece of sweet potato (7 × 7 × 7 mm in size) attached to a needle tip in a cylindrical tube (20 mm in diameter), for 20 trials. The cylindrical tube was located at shoulder height and at a sagittal distance of 150 mm from the cage. Successful retrieval was defined as the monkey retrieving the sweet potato and bringing it to its mouth within 30 s and without dropping it. The proportion of successful retrievals was determined.

### Pharmacological inactivation experiment

The region where hemodynamic responses increased most during functional recovery after the infarcts was inactivated by using muscimol and a procedure similar to that used in our previous studies^21,50^.

The sites of muscimol injection were determined from the results of the fNIRS measurement. For Monkey 1, muscimol was injected into the center of two measurement channels in which the hemodynamic response evoked by the small-object-retrieval task dramatically increased after the recovery of hand movements. The effects of muscimol injection on hand performance were evaluated by using the Klüver board task. The test session to evaluate hand performance was conducted between 40 and 60 min after the muscimol injection. It consisted of 20 trials for each well size (10, 11, and 20 mm in diameter) for each hand, i.e., 120 trials in total. For Monkey 2, the measurement channel in which the hemodynamic response increased most dramatically after the recovery was located in the PMv in the hemisphere contralateral to the infarcts. We injected muscimol into this location and also into the corresponding location in the ipsilateral hemisphere, and we then compared the effects of inactivation of the PMv in each hemisphere. Unfortunately, Monkey 2 did not perform the Klüver board task in a sitting position, probably because it was more tired of sitting in the primate chair for a long period under the effect of muscimol. Indeed, this monkey was able to perform small-object retrieval when it was returned to its cage. Therefore, we evaluated the effect of inactivation with the vertical slit task. Twenty trials were conducted for each hand in the 40 to 60 min after muscimol injection. For both monkeys, an equivalent volume of vehicle (0.1 M phosphate buffer at pH 7.4) was also injected into the same injection sites. The muscimol and vehicle injections were performed three times with intervals of at least 2 days, because the effects of muscimol can persist until the day after injection.

### Histology

The brain damage caused by the infarcts and the locations of the muscimol injections were confirmed histologically in Nissl-stained brain sections as in our previous studies^21,50^. Tissue preparation was performed as previously described^51–54^. Images of Nissl-stained sections were acquired with a microscope (BX60, Olympus, Tokyo, Japan) equipped with a 3CCD color video camera (DHC-950, Sony, Tokyo, Japan) and digitized with an image analysis system (version 2019.1.1, Stereo Investigator, MBF Bioscience Inc.).

### Statistical analysis

The fNIRS data from the 150 trials (75 right hand, 75 left hand) were time-locked (t = 0) to the onset of food retrieval movement for that trial. Then the values of ∆HbO and ∆HbR for each time point for each channel for each monkey were statistically analyzed by using a paired *t*-test between the left-hand trials and right-hand trials, as in our previous study^25^. The differences in the ∆HbO and ∆HbR values between the fNIRS data sets of the left-hand trials and right-hand trials before and after infarction were analyzed by two-way multivariate analysis of variance (two-way MANOVA). Total Hb is also an important index corresponding to the regional cerebral blood volume; however, its change is smaller and less responsive to task execution than ∆HbO and ∆HbR, because HbO and HbR in the functional response usually change in opposite directions to each other. Therefore, for purposes of simplification, we did not focus on the total Hb change. The statistical significance of the difference in peak times of hemodynamic response between the PMv and M1, as well as between each time point, was assessed with the Tukey–Kramer test after one-way ANOVA, and that in the behavioral changes after muscimol injection was assessed with the two-sided Fisher’s exact test. All statistical analyses were performed by using the programming language R (version 3.5.1, R Core Team (2018). R: A language and environment for statistical computing. R Foundation for Statistical Computing, Vienna, Austria. URL http://www.R-project.org.)^55^ with Rstudio software (version 1.2.1335, RStudio, Inc., Boston, MA, USA). Tiled graphs of the time course for each statistical analysis were created with the software Igor Pro (version 8, WaveMetrics, Inc., Lake Oswego, OR, USA). The calculation of simulated light propagation and creation of the spatial activation maps overlapping with anatomical images were performed with Matlab (R2018a, The Mathworks, Inc., Natick, MA, USA).

## Supplementary information

Supplementary Information.
